# Spleen-Dependent Immune Protection Elicited by CpG Adjuvanted Reticulocyte-Derived Exosomes from Malaria Infection Is Associated with Changes in T cell Subsets' Distribution

**DOI:** 10.3389/fcell.2016.00131

**Published:** 2016-11-16

**Authors:** Lorena Martín-Jaular, Armando de Menezes-Neto, Marta Monguió-Tortajada, Aleix Elizalde-Torrent, Míriam Díaz-Varela, Carmen Fernández-Becerra, Francesc E. Borras, Maria Montoya, Hernando A. del Portillo

**Affiliations:** ^1^ISGlobal, Barcelona Centre for International Health Research, Hospital Clínic - Universitat de BarcelonaBarcelona, Spain; ^2^REMAR-IVECAT Group, Germans Trias i Pujol Health Science Research InstituteBadalona, Spain; ^3^Centre de Recerca en Sanitat Animal, Institut de Recerca i Tecnologia Agroalimentàries, Universitat de BarcelonaBarcelona, Spain; ^4^Virology, Pirbright InstitutePirbright, UK; ^5^Catalan Institution for Research and Advanced StudiesBarcelona, Spain

**Keywords:** reticulocyte-derived exosomes, vaccine, malaria, spleen, PD-1 cells, effector memory T-cells, pD1-cells

## Abstract

Reticulocyte-derived exosomes (*rex*) are 30–100 nm membrane vesicles of endocytic origin released during the maturation of reticulocytes to erythrocytes upon fusion of multivesicular bodies with the plasma membrane. Combination of CpG-ODN with rex obtained from BALB/c mice infected with the reticulocyte-prone non-lethal *P. yoelii* 17X malaria strain (*rexPy*), had been shown to induce survival and long lasting protection. Here, we show that splenectomized mice are not protected upon *rexPy*+CpG inmunizations and that protection is restored upon passive transfer of splenocytes obtained from animals immunized with *rexPy*+CpG. Notably, *rexPy* immunization of mice induced changes in PD1^−^ memory T cells with effector phenotype. Proteomics analysis of *rexPy* confirmed their reticulocyte origin and demonstrated the presence of parasite antigens. Our studies thus prove, for what we believe is the first time, that *rex* from reticulocyte-prone malarial infections are associated with splenic long-lasting memory responses. To try extrapolating these data to human infections, *in vitro* experiments with spleen cells of human transplantation donors were performed. Plasma-derived exosomes from vivax malaria patients (*exPv*) were actively uptaken by human splenocytes and stimulated spleen cells leading to changes in T cell subsets.

## Introduction

Extracellular vesicles are cell-secreted lipid bilayer structures that can be classified based on their size and subcellular origin in two major types: exosomes and microvesicles (Raposo and Stoorvogel, 2013). Exosomes are small membrane vesicles (30–100 nm) of endocytic origin; thus, an internalized segment of plasma membrane generates multivesicular bodies containing small vesicles that are released after fusion with the membrane. Exosomes were initially described as a mechanism for removal of selected unwanted proteins in the maturation of reticulocytes to erythrocytes (Harding et al., 1983; Pan and Johnstone, 1983) Subsequent studies demonstrated that several additional cell types secreted exosomes (Raposo et al., 1996; Théry et al., 2009). Moreover, it is commonly accepted that these vesicles can carry biological signals, such as proteins and miRNAs and are implicated in intercellular communication without direct cellular contact (Valadi et al., 2007; Théry et al., 2009; Raposo and Stoorvogel, 2013; Yáñez et al., 2015)

Exosomes in malaria were described for the first time in the rodent model of BALB/c mice infected with *Plasmodium yoelii* 17X (Martin-Jaular et al., 2011). Of note, in this non-lethal malaria murine model, the parasites present a tropism for reticulocytes, the original cells from which exosomes were described in rats (Harding et al., 1983; Johnstone et al., 1987). Subcutaneous immunization of mice with exosomes isolated from *P.yoelii*-infected reticulocytes (*rexPy*) in combination with CpG-ODN elicited IgG antibodies with capacity of recognizing iRBCs. Furthermore, *rexPy* immunization promoted the survival and subsequent long lasting protection of animals lethally challenged (Martin-Jaular et al., 2011). These results demonstrated that *rex* were able to activate specific protective immune responses.

During the blood stages of malaria infection, the spleen is the main organ involved in elimination of iRBCs and the development of the immune responses (Engwerda et al., 2005; Del Portillo et al., 2012). In spite of this key role, very little is known about immune responses elicited in the spleen in malaria even though red pulp macrophages have been shown to have a central role in iRBCs clearance (Yadava et al., 1996). This fact is differential from infections caused by viruses and bacteria where pathogens are destroyed at the marginal zone. It has been suggested that some iRBCs arrive to the marginal zone allowing the capture of parasite antigens by macrophages or migrating dendritic cells from this part of the spleen (Engwerda et al., 2005). Regardless of the site of antigen presentation, once it occurs, both antibodies and CD4^+^ T cells are known to be critical components of protection against blood-stage *Plasmodium* parasite infections (Cohen et al., 1961; Kumar and Miller, 1990). However, other studies strongly suggest that CD8^+^ T cells have also a key role in protection against chronic blood-stage malaria (Imai et al., 2010; Horne-Debets et al., 2013), a finding also found in early studies (Podoba and Stevenson, 1991). Furthermore, recent research in malaria has established that CD4^+^ and CD8^+^T cells experience exhaustion, a dysfunction of T-cells preventing optimal control of chronic infections (Chandele et al., 2011; Butler et al., 2012; Horne-Debets et al., 2013; Illingworth et al., 2013). Programmed cell death-1 receptor (PD-1) mediated lymphocyte exhaustion leads to poor effector functions and loss of immune protection, and could be thus the reason of the lack of lasting immunity against malaria (Wykes et al., 2014).

To better understand the molecular basis of the *rexPy*-induced protection, we have now analyzed spleen and T cell responses to *rex* in BALB/c mice and determined the molecular composition *rexPy*. To extrapolate results to humans, *in vitro* T cell responses of splenocytes obtained from transplantation donors were performed.

## Materials and methods

### Mice and parasites

All the animal studies were performed at the animal facilities of Hospital Clinic in Barcelona in accordance with guidelines and protocols approved by the Ethics Committee for Animal Experimentation of the University of Barcelona CEEA-UB.

Female BALB/c mice, 7 to 9 weeks of age, were used throughout the study. Splenectomized BALB/c mice were obtained from Charles River Laboratories.

The Plasmodium yoelii non-lethal strain 17XNL(1.1) (MRA-593) and the P. yoelii lethal strain 17XL (MRA-680) were obtained from MR4, ATCC® Manassas Virginia. Infections were maintained in Balb/c mice by intraperitoneally (i.p.) injection of 5 × 10^5^pRBCs from the tail blood of donor mice at 5–10% parasitemia. Parasitemia was monitored by Giemsa staining of blood smears.

### Immunizations and challenge

For immunizations, mice were injected subcutaneously (s.c.) with 10 μg of exosomes and 10 μg CpG ODN-1826. Twenty days after, mice were re-immunized with 5 μg of exosomes. Twenty days after the second immunization, mice were analyzed for spleen cellular responses. In challenge experiments, mice were infected with 5 × 10^5^ *P. yoelii* 17XL 20 days after the second immunization. Parasitemia was followed using Giemsa-stained blood smears.

### Splenocyte transfer

Splenocytes were obtained from the spleens of mice immunized with *rexC* and *rexPy* on day 20. Briefly, the spleens were homogenized and passed through a nylon mesh to create a single-cell suspension. Recipient mice in transfer experiments received 10^8^ splenocytes re-suspended in 500 μL of phosphate-buffered saline (PBS) by injection into the tail vein.

### Purification of reticulocytes

Reticulocytes were obtained from mice blood collected in EDTA. Blood from non-infected mice or mice infected with *P. yoelii* 17X strain at 20–30% parasitemia was obtained by intracardiac puncture and passed through a CF11 cellulose filter to remove the leukocyte population (Venkatesan et al., 2012). Reticulocytes were purified by layering them on top of a Percoll/NaCl gradient (1.058–1.096 g/mL). After 250 × g centrifugation for 30 min at 4°C, reticulocytes were collected from the interface of the two Percoll layers. Purified reticulocytes were washed twice and cultured for 24 h at 37°C in DMEM, supplemented with 5 mM glutamine, 5% fetal calf serum, 50 U/mL penicillin, and 50 g/mL streptomycin at 1–3% hematocrit. We obtained 7 ± 3 × 10^7^ reticulocytes from uninfected mice and 3,6 ± 0,6 × 10^8^ reticulocytes from per *P. yoelii* 17X-infected mice. To remove exogenous exosomes in the culture medium, the fetal calf serum was precentrifuged (100,000 × g overnight).

### Exosomes purification

Exosomes from supernatant of reticulocyte cultures were purified by sequential centrifugations at 500 × *g* for 30 min, 20,000 × *g* for 45 min, and 100,000 × *g* for 2 h at 4°C. The final pellet was resuspended in PBS, filtered through a 0.22-μm membrane, and centrifuged at 100,000 × *g* for 2 h at 4°C. The pellets were resuspended in PBS, and the protein content was determined by Bradford assay. Routinely, exosomes purity was assessed by ME analysis as previously described (Martin-Jaular et al., 2011). Exosomes from plasmas of *P. vivax* patients were obtained with the same differential centrifugation method.

### T cell phenotypes

Spleen cells were obtained from non-immunized mice, self-cured mice 25 days post *P. yoelii* 17X infection and immunized mice with *rexPy* or *rexC* on day 20 after the second immunization, as described above. Splenocytes were analyzed for the expression of different markers with an LSRFortessa flow cytometer and data were analyzed with FlowJo software. T cells subsets were identified according to lymphocytes' SSC-A/FSC-A profile and labeling with CD4-PerCp or CD8-Alexa Fluor-700 antibodies (Biolegend). The phenotype of CD4^+^ and CD8^+^T cells in spleen was analyzed with a panel of fluorochrome-conjugated antibodies obtained from Biolegend that included: CD62L-Pacific Blue, PD1-PE/Cy7, CD127-PE and CD44-FITC.

### Mass spectrometry

An LTQ OrbitrapVelos (Thermo Fisher) was used for performing liquid Chromatography (nanoLCULTRA-EKSIGENT) followed by mass spectrometry (nanoLC-MS/MS). Samples of isolated vesicles (rexPy) were reduced with 10 mM DTT (Dithiothreitol), alkylated with 55 mM iodoacetamide, precipitated by 10% TCA, washed with 100% acetone and reconstituted in 2 μL of 8M urea. Samples were brought to a concentration of 1.6 M urea, 1 μg of trypsin (*Sus scrofa*) was added and digestion was carried overnight at 37°C. The reaction was stopped with 1% formic acid. Trypsinized samples were injected into a precolumn (C18PepMap-100-Thermoscientific-5 mm-ID300 um-5 um-100A) before being flushed into the analytical column (AcclaimPepMap100-Thermoscientific-15 cm-ID75 um-3 um-100A-C18) and eluted at a flow rate of 400 nL/min with a mobile phase gradient: 0–40% of solvent B in solvent A in the first 80–90 min and then 40–100% of solvent B in solvent A until the finish of the run at 110–120 min (A: 3% Acetonitrile 0.1% Formic acid in water, B: 97% Acetonitrile 0.1% Formic acid in water). The eluate was applied to the nanospray source of the Orbitrap spectrometer and a full scan was acquired for all spectra within the 400–1500 m/z range with a 30.000 resolution and a maximum injection time of 500 ms. The MS/MS was performed in the LTQ and the top 20 most intense peptides were isolated and fragmented by low energy CID, 35% collision energy.

### Database search and protein identification

Raw spectral data from Xcalibur™ (Thermo Scientific, v2.1) was searched against a custom database compiled from (i) the RefSeq mouse (*Mus musculus*) reference proteome with isoforms (51193 entries, downloaded from www.uniprot.org on 25th October 2013), (ii) the predicted proteome of *Plasmodium yoelii* (5979 entries, downloaded from www.genedb.org in its 2nd version), (iii) the RefSeq reference proteome for *Bos taurus* (24210 entries downloaded from www.uniprot.org on 18th February 2014), (iv) trypsin from *Sus scrofa* (Accession P00761 downloaded from www.uniprot.org on 14th October 2013), and (v) keratins from the RefSeq reference proteome for *Homo sapiens* (236 proteins downloaded from www.uniprot.org on 18th July 2013), totaling 81619 proteins. The predicted proteome for *Bos taurus* was added to the database to control for the presence of proteins from the culture medium, even though only exosome-depleted FBS was used in the preparation of culture media. The search was done with the Sequest HT algorithm on the Proteome Discoverer™ Software version 1.4.1.14 (Thermo Scientific). Searches were performed with the following parameters: digestion by trypsin, 2 missed cleavage sites allowed, precursor mass tolerance of 10 ppm, fragment mass tolerance of 0.6 Da, oxidation of methionine as the variable modification and carbamidomethylation of cysteine as the fixed modification. The Signal-to-Noise (S/N) threshold was set to 1.5. Percolator was used for PSM validation at 1% false discovery rate (FDR) at peptide level. High confidence peptides (1% FDR) were further filtered at ΔCn ≤ 0.1 and Xcorr greater than 1.5, 2.0, 2.25, 2.5, 2.75, 3.0, 3.2, and 3.4 for charge states 1, 2, 3, 4, 5, 6, 7, and > 7, respectively. Proteins identified by the same set of peptides were grouped under one master protein entry. Finally, proteins detected by two or more peptides and present in at least 2 (out of 4) preparations were considered as the core proteomes, for mouse or parasite, from the infected reticulocyte-derived exosomes (*rexPy*). “The mass spectrometry proteomics data have been deposited to the ProteomeXchange Consortium via the PRIDE partner repository with the dataset identifier PXD005048.”

### Go terms over-representation analysis

The Biological Networks Gene Ontology tool–BiNGO (Maere et al., 2005) was used to perform the analysis of over represented GO terms among the detected proteins in the core proteomics set. The reference ontology file used (*go.obo*, version 1.2 release-date 26/01/2015) was downloaded from www.geneontology.org. The annotation file for *Mus musculus* was custom made from the *gene_association.mgi* file (downloaded from www.geneontology.com, release-date 22/01/2015) and the annotation file for *Plasmodium yoelii* was custom made using an in-house script to parse GO data downloaded from for the *Plasmodium yoelii* 17X strain from www.plasmodb.org (version 13.0). The analyses were performed with the Hypergeometric test, using the Benjamini and Hochberg False Discovery Rate correction for multiple testing. *P*-values under 0.05 were considered as significant.

### Human spleen cells

Human spleens were retrieved from deceased donors between 18 and 60 years old after brain death or circulatory death at the Hospital Clínic of Barcelona in accordance with a protocol approved by the Ethics Committee for Clinical Research of the University of Barcelona (No 041499). Neoplasms affecting the spleen or concomitant infection were exclusion criteria. The organ was conserved in saline solution. A spleen portion of about 4 g was homogenized with a GentleMACS™. After that, the sample was washed and resuspended in RPMI medium. The resulting volume was filtered in a 70 μm nylon cell strainer to obtain single cell suspensions.

### Exosome labeling

*exPv* were labeled using the PKH67 labeling midi kit (Sigma) according to the manufacturer's protocol with minor modifications. Briefly, 10–50 μg of exosomes diluted in PBS were added to 0.5 ml of Diluent C. 2 μl of PKH67 dye was added to 0.5 ml of Diluent C before being added to the exosomes. The samples were mixed gently for 5 min and labeling was stopped by addition of 2 ml of exosome-free FBS for 2 min, followed by the addition of PBS to fill up the centrifuge tube. Labeled exosomes were washed by ultracentrifugation for 90 min at 100,000 × *g*. After two additional washes in PBS to remove residual lipid dye, the PKH67 exosomes were resuspended in PBS. Each exosome preparation was stored at 4°C and used within 2 days after labeling.

### Uptake of exosomes by spleen cells

10^6^ splenocytes resuspended in DMEM containing 5% exosome-depleted FBS were seeded on a 24-well plate. Plates were incubated at 37°C, 5% CO_2_, 100% humidity for 1 h. Exosomes labeled with fluorescent PKH67 (5 μg/ml) were added to spleen cells and incubated at 37 or 4°C for different times. Cells were then washed three times with PBS and analyzed on an LSRFortessa flow cytometer and data were analyzed with FlowJo software (Tree Star Inc).

### Exosome stimulation of human spleen cells

5 × 10^5^ splenocytes obtained from human donors were incubated with medium alone or medium containing exosomes from plasma of *P. vivax* patients (10 μg/ml). Cells were cultured at 37°C in RPMI supplemented with 10% fetal calf serum, 50 U/mL penicillin, and 50 μg/mL streptomycin. After 72 h, cells were labeled with a panel of antibodies in order to discriminate different types of cells (Figure S1) and analyzed in an LSRFortessa flow cytometer.

## Results

### Essential role of the spleen in *rexPy* induced protection

**Figure 1:**
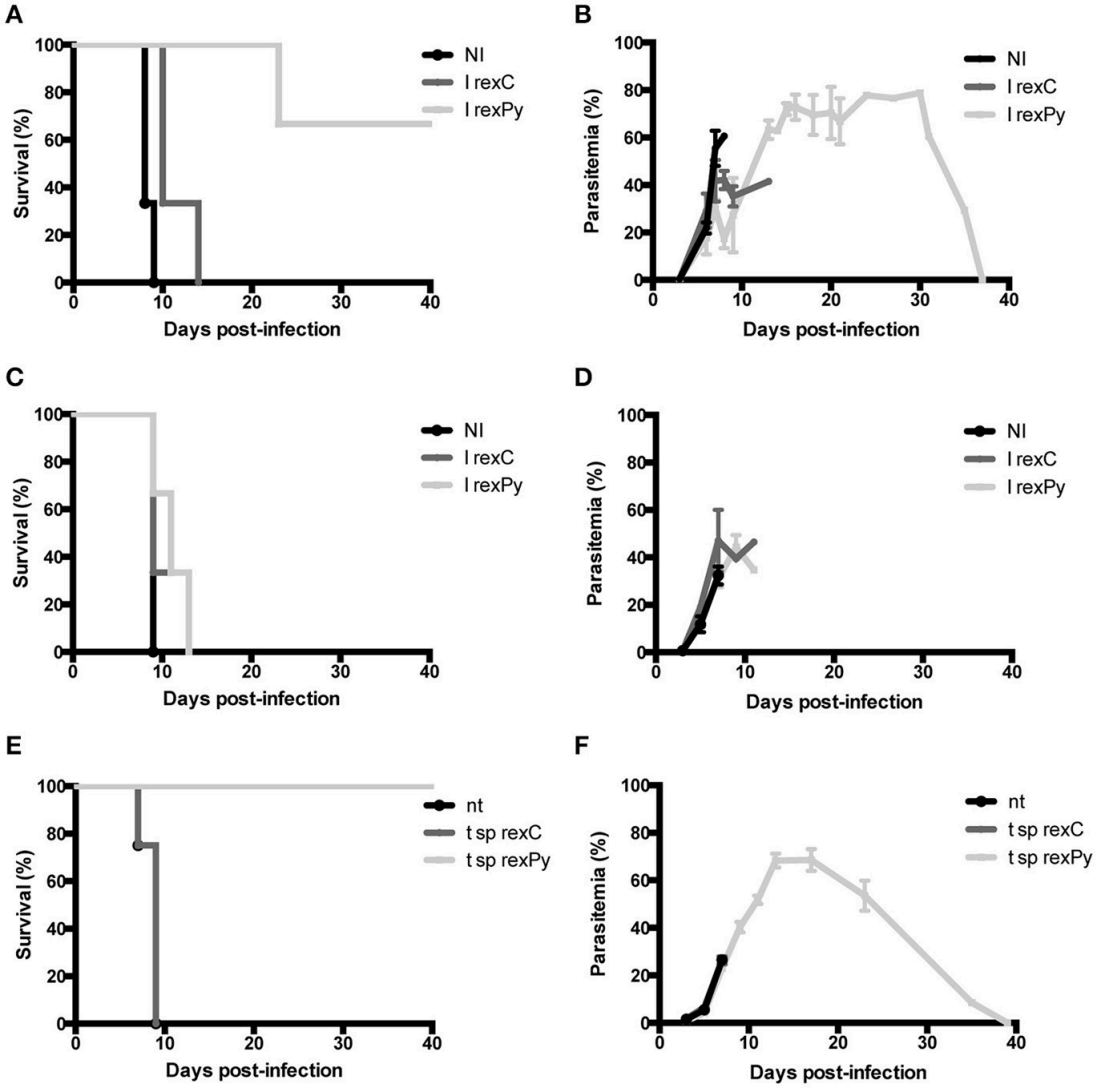
**Protection of BALB/c mice upon immunization with rexPy + CpG-ODN and lethal challenge is spleen-dependent. (A)** Survival curve and **(B)** daily time parasitemia after 5 × 10^5^ *P. yoelii* 17XL infections of groups of BALB/c previously immunized with subcutaneously (s.c.) with CpG-ODN-1826 plus *rex*C (*n* = 3) or *rex*Py (*n* = 3). Non-immunized (NI) mice (*n* = 3) were untreated. Differences in the survival curves between NI, I *rex*C and I *rexPy* (*P* < 0.05) are statistically significant [Log-rank (Mantel-Cox Test)]. Parasitemia is represented as mean + SD of animals with presence of parasite in blood. I *rex*C mice were recovered (no parasite in blood) at days 13,23 and 37. **(C)** Survival curve and **(D)** daily time-course parasitemia after 5 × 10^5^ *P. yoelii* 17XL infections of groups of splenectomized BALB/c mice previously immunized subcutaneously (s.c.) with CpG-ODN plus *rex*C (*n* = 3) or *rex*Py (*n* = 3). Non-immunized (NI) mice (*n* = 3) were untreated. Parasitemia is represented as mean + SD of animals with presence of parasite in blood. **(E)** Survival curve and **(F)** daily time-course parasitemia after 5 × 10^5^ *P. yoelii* 17XL infections of groups of animals previously transferred with splenocytes from *rexC* (t sp*rexC, n* = 4) and *rexPy* (t sp *rexPy, n* = 4) immunized animals. Non-transferred (nt) mice (*n* = 4) were untreated. Differences in the survival curves between nt and t sp *rexPy* (*P* < 0.05) and between t sp *rex*C and t sp *rexPy* (*P* < 0.05) are statistically significant [Log-rank (Mantel-Cox Test)]. Parasitemia is represented as mean + SD of animals with presence of parasite in blood. I *rex*C mice were recovered (no parasite in blood) at days 23,33 (*n* = 2) and 39.

We have previously described a *rexPy* plus CpG-ODN vaccination approach that induced survival after first *P. yoelii* 17XL infection in immunized BALB/c mice and long-lasting sterile protection in subsequent challenges (Martin-Jaular et al., 2011). To analyze whether the protection was mediated by the spleen, groups of normal (Figures 1A,B) or splenectomized BALB/c mice (Figures 1C,D) were immunized subcutaneously (s.c.) with 10 μg of reticulocyte-derived exosomes from non-infected BALB/c mice (*rexC*) or *rexPy* combined with 10 μg CpG-ODN. Twenty days after the first immunization, mice were boosted s.c. with 5 μg of exosomes. Non-immunized mice (NI) were left untreated. Twenty days after the second immunization, all mice were infected with 5 × 10^5^ *P. yoelii* 17XL lethal parasites and parasitemia was monitored. Protection was achieved in mice immunized with *rexPy* (Figures 1A,B) but did not occur in splenectomized animals (Figures 1C,D), indicating that the spleen played an essential role in the protective immune responses induced by these exosomes.

To address the role of spleen cells in protection from the lethal challenge, spleens were collected from *rexC* and *rexPy* immunized mice 2 weeks after the second immunization and cells were adoptively transferred into naïve animals. Groups of 4 naïve BALB/c mice were transferred with splenocytes as described in the methods section. Recipient animals were challenged with 5 × 10^5^ *P. yoelii* 17XL lethal parasites 24 h post-transfer and parasitemia monitored. A group of non-transferred mice were used as controls of experimental lethal infections. The animals treated with splenocytes obtained from *rexPy-*immunized animals were protected from lethal challenge (Figures 1E,F). In contrast, none of the animals receiving splenocytes from untreated animals, nor splenocytes from animals immunized with *rexC*+CpG, survived the lethal challenge. These data strongly suggested that protection was mediated by splenic cellular immunity.

### *rexPy*+CpG immunization lead to increase of PD1−memory CD62L−cells in spleen

**Figure 2:**
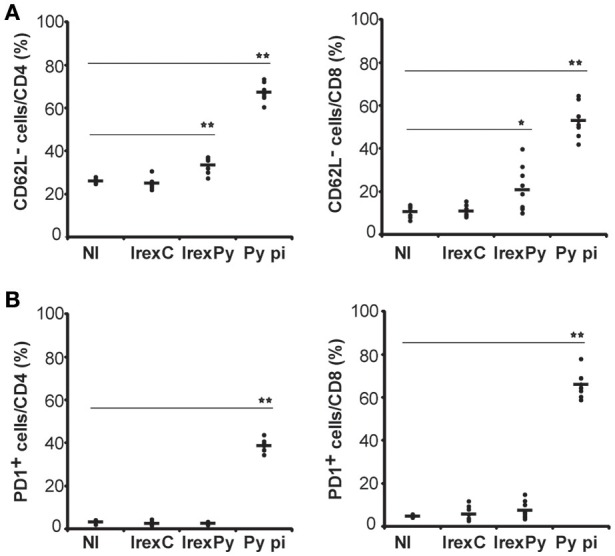
**Analysis of spleen T-cells after immunization with exosomes**. BALB/c mice were immunized with *rexC* or *rexPy* plus CpG-ODN-1826. Two weeks after the second immunization spleen cells were obtained. Non-immunized mice (NI) were untreated. A group of mice was infected with *P. yoelii* 17X and spleen cells were obtained 25 days post infection (Py p.i). Spleen cells were labeled with the panel of antibodies described in materials and methods and analyzed in LSRFortessa. **(A)** Quantification of the percentage of CD4^+^ or CD8^+^ CD62L^−^ cells. **(B)** Quantification of the percentage of CD4^+^ or CD8^+^PD1^+^ cells. **(A,B)** Bold line corresponds to the mean of 6 to 9 mice per group from three different experiments. Percentages are evaluated with analysis of variance and vs.non-immunized group (Dunnet *post hoc test*). (^*^P < 0.05, ^**^P < 0.01).

Having demonstrated that passive transfer of splenocytes from animals immunized with *rexPy*+CpG conferred full protection against lethal challenge, we evaluated whether exosomes were able to promote the appearance of memory cells in the spleen. Therefore, splenocytes from immunized mice were analyzed 20 days after the second immunization to evaluate the phenotype of the cells elicited by our vaccination protocol. In order to compare the immune response to *rexPy*+CpG vaccination with immunity to malaria infection, a group of animals were infected with *P. yoelii* 17X causing a self-resolving reticulocyte-prone infection and analyzed after clearance of parasitemia. Splenocytes from immunized and self-cured mice were stained for different surface markers in order to discriminate between different populations by flow cytometry and percentages of different subsets of T cells were evaluated. We found that percentages of both CD4^+^CD62L^−^ and CD8^+^CD62L^−^cells were increased in spleens of mice immunized with *rexPy* when compared with mice immunized with *rexC* or non-immunized animals. Similar results, *albeit* of higher magnitude, were observed in non-immunized animals recovered from *P. yoelii*17X experimental infections (Figure 2A). However, after self-cured parasite infection, around 40% of CD4^+^T cells and 60% of CD8^+^T cells expressed the exhaustion marker PD-1, indicating loss of their functional capacity. Percentages of PD-1^+^cells in vaccinated animals were not statistically different from those in mice immunized with *rexC* or non-immunized animals (Figure 2B).

**Figure 3:**
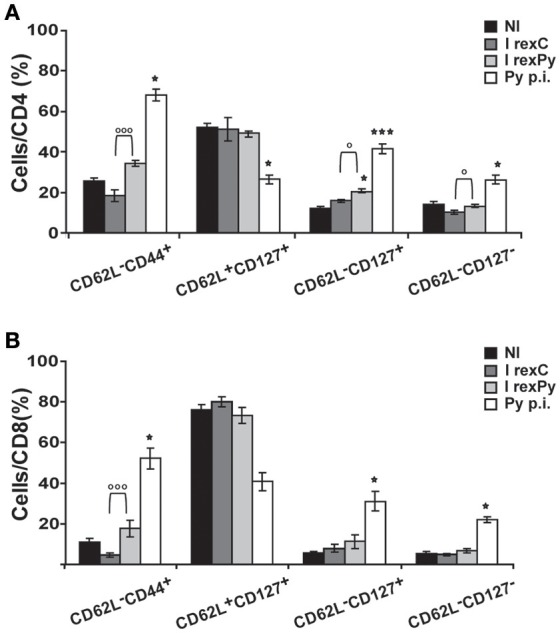
**Phenotypic analysis of T-cells after immunization with exosomes**. BALB/c mice were immunized with *rexC* or *rexPy* plus CpG-ODN-1826. Two weeks after the second immunization spleen cells were obtained. Non-immunized mice (NI) were untreated. A group of mice was infected with *P. yoelii* 17X and spleen cells were obtained 25 days post infection (Py p.i). Spleen cells were labeled with the panel of antibodies described in material and methods and analyzed in a LSR Fortessain order to differentiate between the different T cell memory populations: effector memory T cells (CD62L^−^CD44^+^), central memory (CD62L^+^CD127^+^), effector memory (CD62L^−^CD127^+^) and activated effector T cells (CD62L^−^CD127^−^). **(A)** Quantification of the percentage of different population subsets from CD4^+^cells. **(B)** Quantification of the percentage of different subsets from CD8^+^cells. Data correspond to the mean ± standard error of 9 mice per group from three different experiments. Percentages are evaluated with Kruskal-Wallis test and *vs*. non-immunized group (Dunn's *post hoc test*). (^*^P < 0.05, ^***^P < 0.001). Differences between I *rexC* and I *rexPy* groups are analyzed with Mann-Whitney test (◦*P* < 0.05, ^◦◦◦^*P* < 0.001).

In order to get insight into T cell subsets after immunization and compared them with the ones after self-cured infection, CD62L and CD127 markers were used in order to differentiate between activated effector (CD62L^−^CD127^−^) and effector memory (CD62L^−^CD127^+^) T cells. Moreover, we investigated the relative contribution of effector memory pools by CD44 and CD62L surface expression. Percentages of all the different populations analyzed were statistically different after a self-cured infection of *P. yoelii* 17X when comparing with non-immunized mice and mice immunized with *rexC* or *rexPy* (Figures 3A,B), indicating differences in the role and relevance of the CD4^+^ and CD8^+^ T cell subsets between natural infection and after exosome immunization. *rexPy* immunization lead to a significant increase in the percentage of effector memory CD4^+^ T cells (CD62L^−^CD127^+^) compared to non-immunized animals or animals immunized with *rexC* (Figure 3A). An increasing trend in CD8^+^ T effector memory cells was also observed in *rexPy*-immunized animals (Figure 3B).

All together, these results confirmed the increase in the percentages of both CD4^+^ and CD8^+^ T CD62L^−^memory cells in the spleens from *rexPy*-immunized mice and after self-resolved infections. Importantly, PD-1^+^ T cells were found only after *P. yoelii*17X infection. Both CD4^+^ and CD8^+^ T cells after *rexPy* immunization had mainly an effector phenotype.

### Reticulocyte-derived exosomes from infections with *P. yoelii* 17x (*rexPy*) contain parasite proteins

Purified *P. yoelii*-infected reticulocytes were obtained by removing all contaminating leukocytes followed by a Percoll gradient. Infected reticulocytes were then culture *in vitro* for 24 h in an exosome-depleted medium. After confirming that the viability was >98%, *rexPy* were obtained from supernatants following a sequential centrifugation/filtration protocol and analyzed by mass spectrometry as described in material and methods.

**Figure 4:**
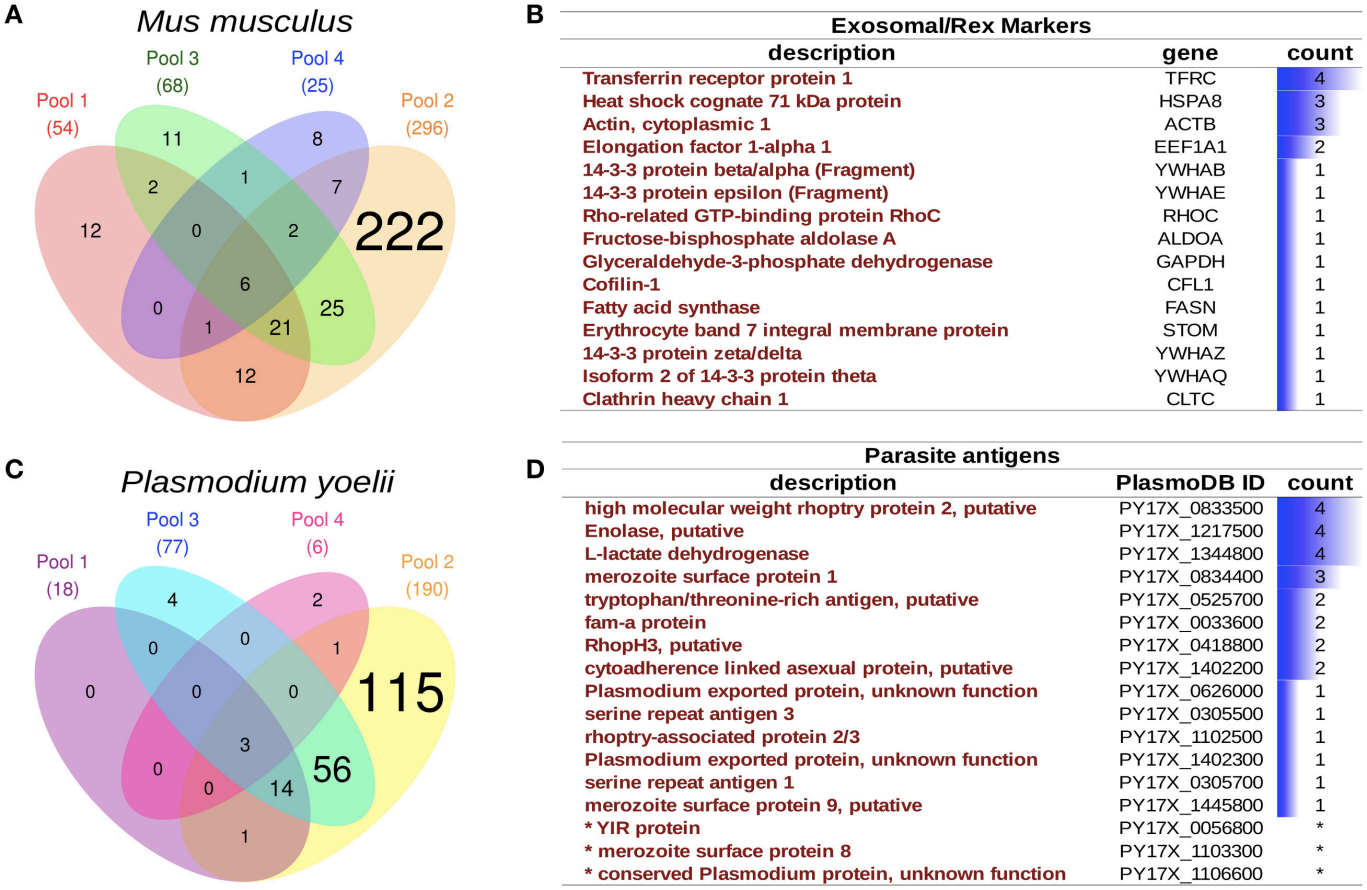
**Proteomics analysis of infected reticulocyte-derived exosomes - ***rexPy***. (A)** *rexPy* were isolated after 48 h cultures of reticulocytes purified from blood of *P. yoelii*-infected BALB/c mice. Four *rexPy* preparations from pooled infected animals (*n* = 3 each) were examined by MS and both host and parasite proteins were detected. Venn diagram depicting mouse proteins identified by MS in each of the four preparations. **(B)** Table of the exosomal/*rex* markers that were detected among the mouse proteins and the number of preparations in which they were detected. **(C)** Venn diagram depicting parasite (*Plasmodium yoelii*) proteins identified by MS in each of the four preparations. **(D)** Table of relevant parasite proteins identified by MS, the asterisk (^*^) denotes proteins that were identified by single-peptides only.

Four *rexPy* preparations from pooled infected animals (*n* = 3 each) were examined by mass spectrometry and both host and parasite proteins were detected. Although the total number of proteins varied among preparations (Figures 4A,C), there were sets of recurrently identified proteins shared by at least two preparations, to, which we denoted as the core *rexPy* proteome. From host origin, the core proteome was composed of 77 proteins (Dataset S1), among which four known exosomal markers were detected. Of interest, the transferrin receptor (TFRC) and the heat shock 70 protein (HSPA8), two proteins classically associated to reticulocyte-derived exosomes since their first description (Johnstone et al., 1987), were detected in 4/4 and 3/4 preparations, respectively (Figure 4B). A 40S ribosomal protein, three hemoglobin subunits (alpha, beta-1, and beta-2) and elongation factor 2 were also detected on all 4 preparations (Dataset S1). Several other exosomal markers (Figure 4B) and some MCH I proteins (Dataset S1) were also detected.

An overrepresentation analysis of GO terms on the *rexPy* core proteome supports the conclusion that the preparations were of vesicular origin as several terms related to extracellular vesicles were significantly overrepresented in the Cellular Component annotation (Dataset S2). Among parasite proteins identified, there was also a variably overlap among preparations (Figure 4C), and the core proteome was composed of 75 proteins. Noticeably, in the list of parasite proteins (Dataset S3), there were several which are exposed by the parasite and, thus, could have antigenic properties, such as rhoptry proteins, serine repeat antigens (SERA), fam-a proteins, YIR proteins, exported proteins of unknown function and the merozoite surface protein 1 (MSP1), which is a “classical” malaria vaccine candidate (Figure 4D). The overrepresentation of GO terms suggested an enzymatic activity linked to the catabolism of proteins by the proteasome complex (Dataset S4). Of interest, enolase and L-lactate dehydrogenase were detected in all four preparations and enolase has been shown to have a “moonlight” function in host-parasite interactions as antibodies against this protein are able to protect mice against infection with *P. yoelii* (Pal-Bhowmick et al., 2007).

### *exPv* are captured by human splenocytes and induced an increase in T cells *In vitro*

**Figure 5:**
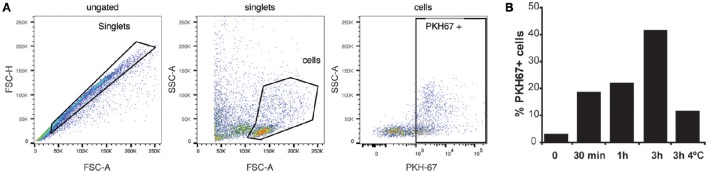
**Uptake of exosomes by human splenocytes**. Exosomes purified from blood of *P vivax* patients were labeled using PKH-67 dye. 10^6^ human spleen cells were incubated with medium (0) or medium containing 5 μg of the PKH67 labeled *exPv* for different times at RT or 4°C to avoid internalization processes. Cells were analyzed in LSR Fortessa flow cytometer. **(A)** Singlet cells were gated according to FSC-A/FSC-H. Incorporation of PKH67-labeled exosomes into spleen cells was analyzed in PKH67/SSC-A plots. **(B)** Percentage of PKH67 positive cells. Data correspond to one representative experiment out of three independent spleen samples.

Infections in BALB/c mice with the *P. yoelii* 17X strain resemble the natural tropism for reticulocytes observed in natural human infections of *P. vivax*, a major human malaria parasite. To try extrapolating the results obtained in this rodent malaria model to human infections by *P. vivax*, experiments with human splenocytes were performed. Worth of mentioning, these cells were obtained from transplantation donors, which limited the experiments. Moreover, due to the absence of an *in vitro* culture system for blood stages of *P. vivax*, exosomes purified directly from plasma of *P. vivax* patients were used. To monitor whether exosomes were captured by human splenocytes, purified *exPv* were labeled with a green fluorescent lipid dye (PKH67) and incubated with spleen cells to assess *exPv* incorporation into splenocytes by flow cytometry (Figure 5A). As expected, when purified PKH-labeled exosomes were incubated with splenocytes for 30 min to 3 h, splenocytes indeed captured *exPv*. Moreover, incubation at 4°C abrogated the incorporation, indicating an active uptake (Figure 5B).

**Figure 6:**
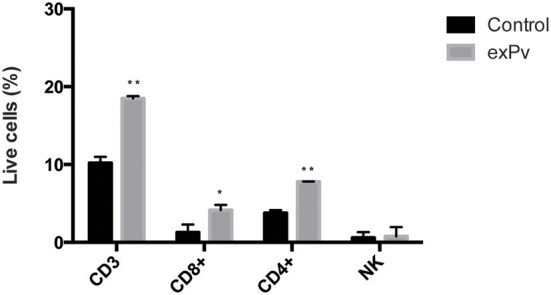
**Stimulation of human splenocytes with exosomes from ***P. vivax*** patients**. Splenocytes obtained from human donors were incubated *in vitro* with medium alone (Control) or medium containing exosomes from plasma of *P. vivax* patients (*exPv*). After 72 h, cells were labeled with a panel of antibodies in order to discriminate different types of cells and analyzed in an LSR Fortessa flow cytometer (Figure S1). Data correspond to three spleen samples and is expressed as mean ± standard error. Numbers of different populations were calculated as a function of singlet cells in the sample. Values are analyzed with the Mann-Whitney test (^*^P < 0.05, ^**^P < 0.01).

To analyze changes in the percentages of different populations of spleen cells in response to exosomes, human splenocytes were incubated with *exPv* for 72 h and cells were labeled with a panel of antibodies to discriminate different subsets of spleen cells by flow cytometry (Figure S1). We found a statistically significant increase of both the number of CD3^+^ T cells and CD8^+^ T cells in response to *exPv* stimulation, while no differences in the number of B or NK cells were observed between splenocytes incubated with exosomes or medium alone (Figure 6). An increasing tendency in the number of CD4^+^ T cells was also observed. In addition, exosomes from healthy donors' plasma (*exC*) were isolated and used as a control of allogeneic stimulation. Incubation with *exC* also incremented CD3^+^ T cell numbers, but to a much lesser extent: a 22% raise compared to the 80% by *exPv* (data not shown).

## Discussion

Here, for what we believe is the first time, we provide data demonstrating that exosomes produced from *Plasmodium yoelii*-infected-reticulocytes (*rexPy*) were associated with the induction of PD1^−^ spleen T-memory cells and protection upon lethal challenge in a rodent malaria model.

The function of reticulocyte-derived exosomes (*rex*) in immune modulation has been poorly explored. Previous work from our group has shown that *rexPy* immunization of BALB/c mice elicits *P. yoelii-specific* IgG antibodies, induces reticulocytosis and changes the cell-tropism from erythrocytes to reticulocytes of the lethal *P. yoelii* 17XL strain (Martin-Jaular et al., 2011). Moreover, long lasting protection was conferred in immunizations of mice with *rexPy* obtained from a reticulocyte *in vitro* culture in combination with CpG-ODN.

In our rex vaccination approach, we found that the spleen plays an essential role in the protective immune response since protection did not occur in splenectomized mice and protection was achieved after splenocyte transfer. However, whether the spleen function is essential either for induction of rex-induced immunity or/and for the effector function during infection remains to be determined. Previous studies have shown that the spleen has a essential role during malaria infection in rodents since splenectomy aggravates self-cured *P. yoelii* 17X and improves *P. yoelii* 17XL infections (Weiss, 1991). The impact of the splenectomy in the course of the *P. yoelii* 17X infection is also dependent on the genetic background of the mouse (Sayles et al., 1991). Moreover, protective immunity to *Plasmodium* parasites can be achieved in susceptible splenectomized mice through adoptive transfer of splenocytes from *P. chabaudi*-infected mice (Yap and Stevenson, 1994). Besides the possible role of the spleen for the resolution of the infection, we have shown that spleen is able to respond to *rexPy* with changes in the populations of T cells and with the increase in the percentages of memory T cells. Future experiments with transfer of specific T cells subsets, antibody-mediated depletion and/or use of deficient mice will need to be done to demonstrate that these cells are responsible for the protection after vaccination.

CD4^+^ and CD8^+^ T cells have a central function in protection against pathogens and in controlling disease progression (Seder et al., 2008). In malaria, the impact of CD4^+^ and CD8^+^ T cell responses in natural infection is partially understood. Thus, it remains a priority to improve the understanding of the role of CD4^+^ and CD8^+^ T cell responses in natural infection as well as in vaccine approaches. For this reason, we analyzed different subsets of T cells in self-cured animals and compared them with vaccinated animals. Phenotypic characteristics of T cells after self-cured *P. yoelii*17X infection differed from those of *rexPy*-immunized animals, indicating differences in immune response between experimental infection and vaccination. *rexPy* vaccination of BALB/c mice promoted CD62L^−^memory T cells increase in the spleen. Moreover, we confirm that the subsets of cells increased by *rexPy* immunization were of effector memory phenotype since the CD4^+^ and CD8^+^ T cell populations increased in the vaccinated group were mainly CD62L^−^CD127^+^ and CD44^+^CD62L^−^. One limitation of these results is that we cannot rule out that total numbers of the different subsets of cells, as opposed to T cell percentages, are not different in these experiments. Yet, no differences in size were recorded among the spleens of the non-immunized, *rexC*-immunized or *rexPy*-immunized animals. Thus, suggesting that the changes observed in the proportion of T cell subsets are minimally affected by differences in cellularity. In addition, our results are in agreement with recent publications showing that CD8^+^ effector memory cells (Reyes-Sandoval et al., 2011; Rigato et al., 2011; Sánchez-Sampedro et al., 2012) and CD4^+^effector memory cells (Sánchez-Sampedro et al., 2012) have a relevant role for long-term protective immunity against parasites in some vaccination approaches as these cells are able to mount a rapid response after encountering the antigen (Colpitts and Scott, 2010). Besides, despite the ethical difficulties of working with human spleen samples, we were able to determine that the number of T cells increased after *exPv* stimulation. This finding highlights the importance of spleen T cells in humans in responding to exosomes.

Evaluation of T-cell exhaustion is important to ensure the development of a long lasting and protective immunological memory response. T cell exhaustion is a T cell dysfunction that appears during many chronic viral infections (Day et al., 2006; Wherry et al., 2007; Ye et al., 2015) and parasite diseases, as well (Gigley et al., 2012). During malaria infection, repeated exposure to antigen drives the expression of T cell inhibitory receptors, including PD-1, and leads to poor effector function (Butler et al., 2012; Gigley et al., 2012; Horne-Debets et al., 2013; Wykes et al., 2014). Thus, we analyzed the PD-1 pathway. Noticeably, self-cured *P. yoelii* 17X infection resulted in PD1 expression in CD4^+^ and CD8^+^ T cells. However, that did not occur in r*exPy-*vaccinated animals, suggesting that cells after vaccination are non-exhausted and are able to perform their protective function. In contrast, exosomes derived from infected erythrocytes have been shown to be pro-inflammatory and have been proposed to contribute to immune cell exhaustion observed during malaria (Mantel et al., 2013). Our data also suggest that *rex* were involved in immune presentation of parasite antigens as protection is only achieved with *rex* from infected hosts. This is a remarkable finding since it suggests that reticulocyte-derived exosomes might represent a novel platform for antigen presentation in malaria capable of inducing long-lasting memory responses.

The MS proteomics analysis of reticulocyte-derived exosomes supports this novel approach for antigen discovery for vaccination. Thus, rhoptry proteins as well as variant surface proteins pertaining to fam-a and YIR proteins were identified as cargo of these exosomes. Rhoptry proteins have an essential role in invasion and variant surface proteins are predicted to have a role in host-parasite interactions. Moreover, MSP1 and enolase have been shown to generate antibodies capable of protecting mice in experimental infections with P. yoelii. Noticeably, reticulocytes express MHC class I molecules required by CD8^+^ T cells for recognition. In fact, H2 K and H2 D antigen levels are increased after infection with *P. yoelii* 17X. Moreover, the level of H2 K expression on *P. yoelii* 17X infected cells from mice of different strains correlates with the ability of each strain to control the infection (Jayawardena et al., 1983). More recently, it has been shown that malaria parasites can parasitize erythroblasts, which have high levels of MHC class I molecules and the capacity to activate CD8+ T cells (Imai et al., 2013). Importantly, H2 K and H2 D molecules were present in *exPy* (Martin-Jaular et al., 2011) and *rexPy* (Dataset S3). Therefore, it might be plausible that exosomes derived from reticulocytes would be able to present parasite antigens both directly and indirectly to T cells. In the absence of experimental data to support this possibility, this remains to be determined.

In conclusion, our results demonstrated that infected reticulocytes-derived exosomes in combination with CpG were able to elicit a spleen-dependent protective response against malaria. This protective response was associated with an increase of the proportion of memory CD4^+^ and CD8^+^ T cells in the spleen, mainly with an effector memory phenotype. On the basis of the results described here, we propose that early presence of non-exhausted effector T cells in the spleen could facilitate the building of a protective and long-lasting immune response against infection. The data presented here thus provide a rational basis for the development of a new vaccine and platform against malaria. This could be particularly relevant for malarial parasites with tropism for reticulocytes, such as *Plasmodium vivax*, the most widely distributed cause of malaria in people (Mueller et al., 2009).

## Author contributions

Conceived the study: LM, HD. Designed the experiments: LM, AD, MMT, CF, FB, MM, HD. Performed the experiments: LM, AD, MMT, AE, MD, and CF. Analyzed the data: LM, AD, MMT, FB, and HD. Wrote the paper: LM, HD.

## Funding

A postdoctoral fellowship from CNPq, ConselhoNacional de DesenvolvimentoCientífico e Tecnologico – Brasil supported AM-N. MMT is sponsored by a Grant (2014FI_B00649) from the “Agència de Gestió d'Ajuts Universitaris i de Recerca” (AGAUR) from the Catalan Government. MDV is a predoctoral fellow supported by “Agència de Gestió d'Ajuts Universitaris i de Recerca” (2015 FI_B 00367). AET is a recipient of a predoctoral fellowship from the Ministerio de Economía y Competitividad, Spain (AP2012-2145). The Ministerio Español de Economía y Competitividad (SAF2012-35133), the European Community's Seventh Framework Programme and Fundación Ramón Areces fund work in the laboratory of HAP.

### Conflict of interest statement

The authors declare that the research was conducted in the absence of any commercial or financial relationships that could be construed as a potential conflict of interest. LM, CF, and HD are named inventors on a patent application covering reticulocyte-derived exosomes as malaria vaccines.

## Supplementary material

Dataset S1 **List of mouse proteins detected by MS/MS**.

Dataset S2 **Over-represented Gene ontology (GO) categories in the core proteome of mouse rexPy**.

Dataset S3 **List of *Plasmodium yoelii* proteins detected by MS/MS**.

Dataset S4 **Over-represented Gene ontology (GO) categories in the core proteome of *Plasmodium yoelii* from mouse rexPy**.

Supplementary Figure 1 **Gating strategy to define human splenocyte populations**. Splenocytes were labeled with a panel of antibodies in order to discriminate immune relevant splenocyte populations (CD3-PE, CD4-APC, CD8-APC-H7, CD45-PerCP, CD16-V450, CD19-Bv510, CD56-PE-Bio770) and analyzed in a BD LSRFortessa flow cytometer. **(A)** Singlet cells were gated according to FSC-A/FSC-W. **(B)** NK cells were determined as CD16^+^/CD56^+^ from singlet population. Two other populations were gated according to CD3 staining: PRE-CD3^+^ and CD3^−^. **(C)** PRE-CD3^+^ cells were confirmed to be lymphocytes by positive CD45 labeling and later classified into the different T cell subsets: CD3^+^/CD4^−^/CD8^−^, CD3^+^/CD4^+^/CD8^−^, CD3^+^/CD4^−^/CD8^+^, or CD3^+^/CD4^+^/CD8^+^. **(D)** CD3^−^ cells were gated as lymphocytes by CD45^+^ staining and B cells were stated as CD19^+^.

## References

[B1] Butler N. S. Moebius J. Pewe L. L. Traore B. Doumbo O. K. Tygrett L. T.. (2012). Therapeutic blockade of PD-L1 and LAG-3 rapidly clears established blood-stage *Plasmodium* infection. Nat. Immunol. 13, 188–195. 10.1038/ni.2180 22157630 PMC3262959

[B2] Chandele A. Mukerjee P. Das G. Ahmed R. Chauhan V. S. (2011). Phenotypic and functional profiling of malaria-induced CD8 and CD4 T cells during blood-stage infection with *Plasmodium yoelii*. Immunology 132, 273–286. 10.1111/j.1365-2567.2010.03363.x 21039472 PMC3050450

[B3] Cohen S. McGregor I. A. Carrington S. (1961). Gamma-globulin and acquired immunity to human malaria. Nature 192, 733–737. 13880318 10.1038/192733a0

[B4] Colpitts S. Scott P. (2010). Memory T-cell subsets in parasitic infections. Adv. Exp. Med. Biol. 684, 145–154. 10.1007/978-1-4419-6451-9_11 20795546

[B5] Day C. L. Kaufmann D. E. Kiepiela P. Brown J. A. Moodley E. S. Reddy S.. (2006). PD-1 expression on HIV-specific T cells is associated with T-cell exhaustion and disease progression. Nature 443, 350–354. 10.1038/nature05115 16921384

[B6] Del Portillo H. A. Ferrer M. Brugat T. Martin-Jaular L. Langhorne J. Lacerda M. V. G. (2012). The role of the spleen in malaria. Cell. Microbiol. 14, 343–355. 10.1111/j.1462-5822.2011.01741.x 22188297

[B7] Engwerda C. R. Beattie L. Amante F. H. (2005). The importance of the spleen in malaria. Trends Parasitol. 21, 75–80. 10.1016/j.pt.2004.11.008 15664530

[B8] Gigley J. P. Bhadra R. Moretto M. M. Khan I. A. (2012). T cell exhaustion in protozoan disease. Trends Parasitol. 28, 377–384. 10.1016/j.pt.2012.07.001 22832368 PMC3768288

[B9] Harding C. Heuser J. Stahl P. (1983). Receptor-mediated endocytosis of transferrin and recycling of the transferrin receptor in rat reticulocytes. J. Cell Biol. 97, 329–339. 6309857 10.1083/jcb.97.2.329 PMC2112509

[B10] Horne-Debets J. M. Faleiro R. Karunarathne D. S. Liu X. Q. Lineburg K. E. Poh C. M.. (2013). PD-1 dependent exhaustion of CD8+ T cells drives chronic malaria. Cell Rep. 5, 1204–1213. 10.1016/j.celrep.2013.11.002 24316071

[B11] Illingworth J. Butler N. S. Roetynck S. Mwacharo J. Pierce S. K. Bejon P.. (2013). Chronic exposure to *Plasmodium* falciparum is associated with phenotypic evidence of B and T cell exhaustion. J. Immunol. 190, 1038–1047. 10.4049/jimmunol.1202438 23264654 PMC3549224

[B12] Imai T. Ishida H. Suzue K. Hirai M. Taniguchi T. Okada H.. (2013). CD8(+) T cell activation by murine erythroblasts infected with malaria parasites. Sci. Rep. 3:1572. 10.1038/srep01572 23535896 PMC3610137

[B13] Imai T. Shen J. Chou B. Duan X. Tu L. Tetsutani K.. (2010). Involvement of CD8+ T cells in protective immunity against murine blood-stage infection with Plasmodium yoelii 17XL strain. Eur. J. Immunol. 40, 1053–1061. 10.1002/eji.200939525 20101613

[B14] Jayawardena A. N. Mogil R. Murphy D. B. Burger D. Gershon R. K. (1983). Enhanced expression of H-2K and H-2D antigens on reticulocytes infected with *Plasmodium yoelii*. Nature 302, 623–626. 6339952 10.1038/302623a0

[B15] Johnstone R. M. Adam M. Hammond J. R. Orr L. Turbide C. (1987). Vesicle formation during reticulocyte maturation. Association of plasma membrane activities with released vesicles (exosomes). J. Biol. Chem. 262, 9412–9420. 3597417

[B16] Kumar S. Miller L. H. (1990). Cellular mechanisms in immunity to blood stage infection. Immunol. Lett. 25, 109–114. 1980907 10.1016/0165-2478(90)90100-5

[B17] Maere S. Heymans K. Kuiper M. (2005). BiNGO: a Cytoscape plugin to assess overrepresentation of gene ontology categories in biological networks. Bioinformatics 21, 3448–3449. 10.1093/bioinformatics/bti551 15972284

[B18] Mantel P.-Y. Hoang A. N. Goldowitz I. Potashnikova D. Hamza B. Vorobjev I.. (2013). Malaria-infected erythrocyte-derived microvesicles mediate cellular communication within the parasite population and with the host immune system. Cell Host Microbe 13, 521–534. 10.1016/j.chom.2013.04.009 23684304 PMC3687518

[B19] Martin-Jaular L. Nakayasu E. S. Ferrer M. Almeida I. C. Del Portillo H. A. (2011). Exosomes from *Plasmodium yoelii*-infected reticulocytes protect mice from lethal infections. PLoS ONE 6:e26588. 10.1371/journal.pone.0026588 22046311 PMC3202549

[B20] Mueller I. Galinski M. R. Baird J. K. Carlton J. M. Kochar D. K. Alonso P. L.. (2009). Key gaps in the knowledge of *Plasmodium vivax*, a neglected human malaria parasite. Lancet Infect. Dis. 9, 555–566. 10.1016/S1473-3099(09)70177-X 19695492

[B21] Pal-Bhowmick I. Mehta M. Coppens I. Sharma S. Jarori G. K. (2007). Protective properties and surface localization of Plasmodium falciparum enolase. Infect. Immun. 75, 5500–5508. 10.1128/IAI.00551-07 17785475 PMC2168313

[B22] Pan B.-T. Johnstone R. M. (1983). Fate of the transferrin receptor during maturation of sheep reticulocytes *in vitro*: selective externalization of the receptor. Cell 33, 967–978. 10.1016/0092-8674(83)90040-5 6307529

[B23] Podoba J. E. Stevenson M. M. (1991). CD4+ and CD8+ T lymphocytes both contribute to acquired immunity to blood-stage *Plasmodium chabaudi* AS. Infect. Immun. 59, 51–58. 1898902 10.1128/iai.59.1.51-58.1991 PMC257704

[B24] Raposo G. Nijman H. W. Stoorvogel W. Liejendekker R. Harding C. V. Melief C. J.. (1996). B lymphocytes secrete antigen-presenting vesicles. J. Exp. Med. 183, 1161–1172. 8642258 10.1084/jem.183.3.1161 PMC2192324

[B25] Raposo G. Stoorvogel W. (2013). Extracellular vesicles: exosomes, microvesicles, and friends. J. Cell Biol. 200, 373–383. 10.1083/jcb.201211138 23420871 PMC3575529

[B26] Reyes-Sandoval A. Wyllie D. H. Bauza K. Milicic A. Forbes E. K. Rollier C. S.. (2011). CD8+ T effector memory cells protect against liver-stage malaria. J. Immunol. 187, 1347–1357. 10.4049/jimmunol.1100302 21715686 PMC4568294

[B27] Rigato P. O. de Alencar B. C. de Vasconcelos J. R. C. Dominguez M. R. Araújo A. F. Machado A. V.. (2011). Heterologous plasmid DNA prime-recombinant human adenovirus 5 boost vaccination generates a stable pool of protective long-lived CD8(+) T effector memory cells specific for a human parasite, *Trypanosoma cruzi*. Infect. Immun. 79, 2120–2130. 10.1128/IAI.01190-10 21357719 PMC3088135

[B28] Sánchez-Sampedro L. Gómez C. E. Mejías-Pérez E. Sorzano C. O. S. Esteban M. (2012). High quality long-term CD4+ and CD8+ effector memory populations stimulated by DNA-LACK/MVA-LACK regimen in *Leishmania major* BALB/c model of infection. PLoS ONE 7:e38859. 10.1371/journal.pone.0038859 22715418 PMC3371028

[B29] Sayles P. C. Cooley A. J. Wassom D. L. (1991). A spleen is not necessary to resolve infections with *Plasmodium yoelii*. Am. J. Trop. Med. Hyg. 44, 42–48. 1996740 10.4269/ajtmh.1991.44.42

[B30] Seder R. A. Darrah P. A. Roederer M. (2008). T-cell quality in memory and protection: implications for vaccine design. Nat. Rev. Immunol. 8, 247–258. 10.1038/nri2274 18323851

[B31] Théry C. Ostrowski M. Segura E. (2009). Membrane vesicles as conveyors of immune responses. Nat. Rev. Immunol. 9, 581–593. 10.1038/nri2567 19498381

[B32] Valadi H. Ekström K. Bossios A. Sjöstrand M. Lee J. J. Lötvall J. O. (2007). Exosome-mediated transfer of mRNAs and microRNAs is a novel mechanism of genetic exchange between cells. Nat. Cell Biol. 9, 654–659. 10.1038/ncb1596 17486113

[B33] Venkatesan M. Amaratunga C. Campino S. Auburn S. Koch O. Lim P.. (2012). Using CF11 cellulose columns to inexpensively and effectively remove human DNA from *Plasmodium falciparum*-infected whole blood samples. Malar. J. 11, 41. 10.1186/1475-2875-11-41 22321373 PMC3295709

[B34] Weiss L. (1991). Barrier cells in the spleen. Immunol. Today 12, 24–29. 10.1016/0167-5699(91)90108-6 2015045

[B35] Wherry E. J. Ha S.-J. Kaech S. M. Haining W. N. Sarkar S. Kalia V.. (2007). Molecular signature of CD8+ T cell exhaustion during chronic viral infection. Immunity 27, 670–684. 10.1016/j.immuni.2007.09.006 17950003

[B36] Wykes M. N. Horne-Debets J. M. Leow C.-Y. Karunarathne D. S. (2014). Malaria drives T cells to exhaustion. Front. Microbiol. 5:249. 10.3389/fmicb.2014.00249 24904561 PMC4034037

[B37] Yadava A. Kumar S. Dvorak J. A. Milon G. Miller L. H. (1996). Trafficking of Plasmodium chabaudi adami-infected erythrocytes within the mouse spleen. Proc. Natl. Acad. Sci. U.S.A. 93, 4595–4599. 8643449 10.1073/pnas.93.10.4595 PMC39322

[B38] Yáñez,-M.ó M. Siljander P. R.-M. Andreu Z. Zavec A. B. Borràs F. E. Buzas E. I.. (2015). Biological properties of extracellular vesicles and their physiological functions. J. Extracell. Vesicles 4:27066. 10.3402/jev.v4.27066 25979354 PMC4433489

[B39] Yap G. S. Stevenson M. M. (1994). Differential requirements for an intact spleen in induction and expression of B-cell-dependent immunity to Plasmodium chabaudi AS. Infect. Immun. 62, 4219–4225. 7927677 10.1128/iai.62.10.4219-4225.1994 PMC303098

[B40] Ye B. Liu X. Li X. Kong H. Tian L. Chen Y. (2015). T-cell exhaustion in chronic hepatitis B infection: current knowledge and clinical significance. Cell Death Dis. 6:e1694. 10.1038/cddis.2015.42 25789969 PMC4385920

