# Corrigendum: Spleen-Dependent Immune Protection Elicited by CpG Adjuvanted Reticulocyte-Derived Exosomes from Malaria Infection Is Associated with Changes in T Cell Subsets' Distribution

**DOI:** 10.3389/fcell.2016.00153

**Published:** 2017-01-17

**Authors:** Lorena Martín-Jaular, Armando de Menezes-Neto, Marta Monguió-Tortajada, Aleix Elizalde-Torrent, Míriam Díaz-Varela, Carmen Fernández-Becerra, Francesc E. Borras, Maria Montoya, Hernando A. del Portillo

**Affiliations:** ^1^ISGlobal, Barcelona Centre for International Health Research, Hospital Clínic-Universitat de BarcelonaBarcelona, Spain; ^2^PVREX and REMAR-IVECAT Groups, Germans Trias i Pujol Health Science Research Institute (IGTP)Badalona, Spain; ^3^Centre de Recerca en Sanitat Animal, Institut de Recerca i Tecnologia Agroalimentàries, Universitat de BarcelonaBarcelona, Spain; ^4^Virology, Pirbright InstitutePirbright, UK; ^5^ICREA, Catalan Institution for Research and Advanced StudiesBarcelona, Spain

**Keywords:** reticulocyte-derived exosomes, vaccine, malaria, spleen, PD-1 cells, effector memory T-cells

Due to an oversight, PVREX was missing from affiliation 2 and ICREA from affiliation 5. The correct affiliations appear above. This does not change the scientific conclusion of the article in any way. The authors apologize for this oversight.

## Conflict of interest statement

The authors declare that the research was conducted in the absence of any commercial or financial relationships that could be construed as a potential conflict of interest.

