# Supplementary figures and images for: Spleen-Dependent Immune Protection Elicited by CpG Adjuvanted Reticulocyte-Derived Exosomes from Malaria Infection Is Associated with Changes in T cell Subsets' Distribution
Source: Front Cell Dev Biol. 2016 Nov 16;4:131. doi: 10.3389/fcell.2016.00131 (PMC5110551; doi:10.3389/fcell.2016.00131)

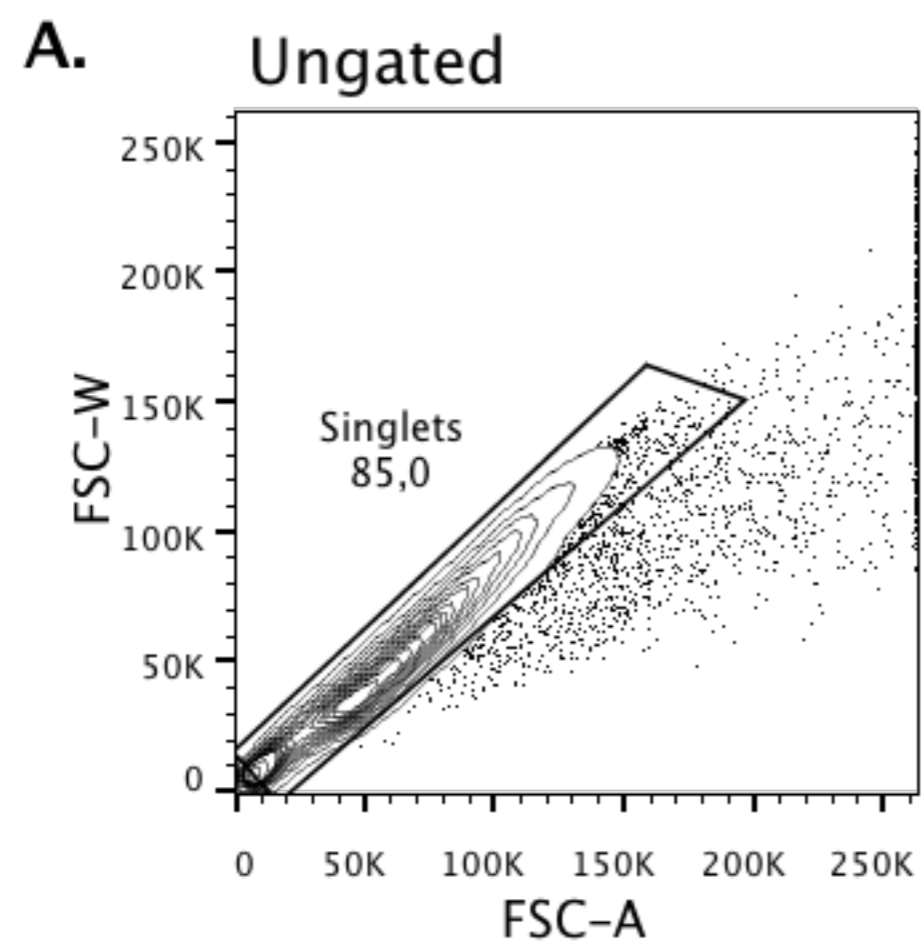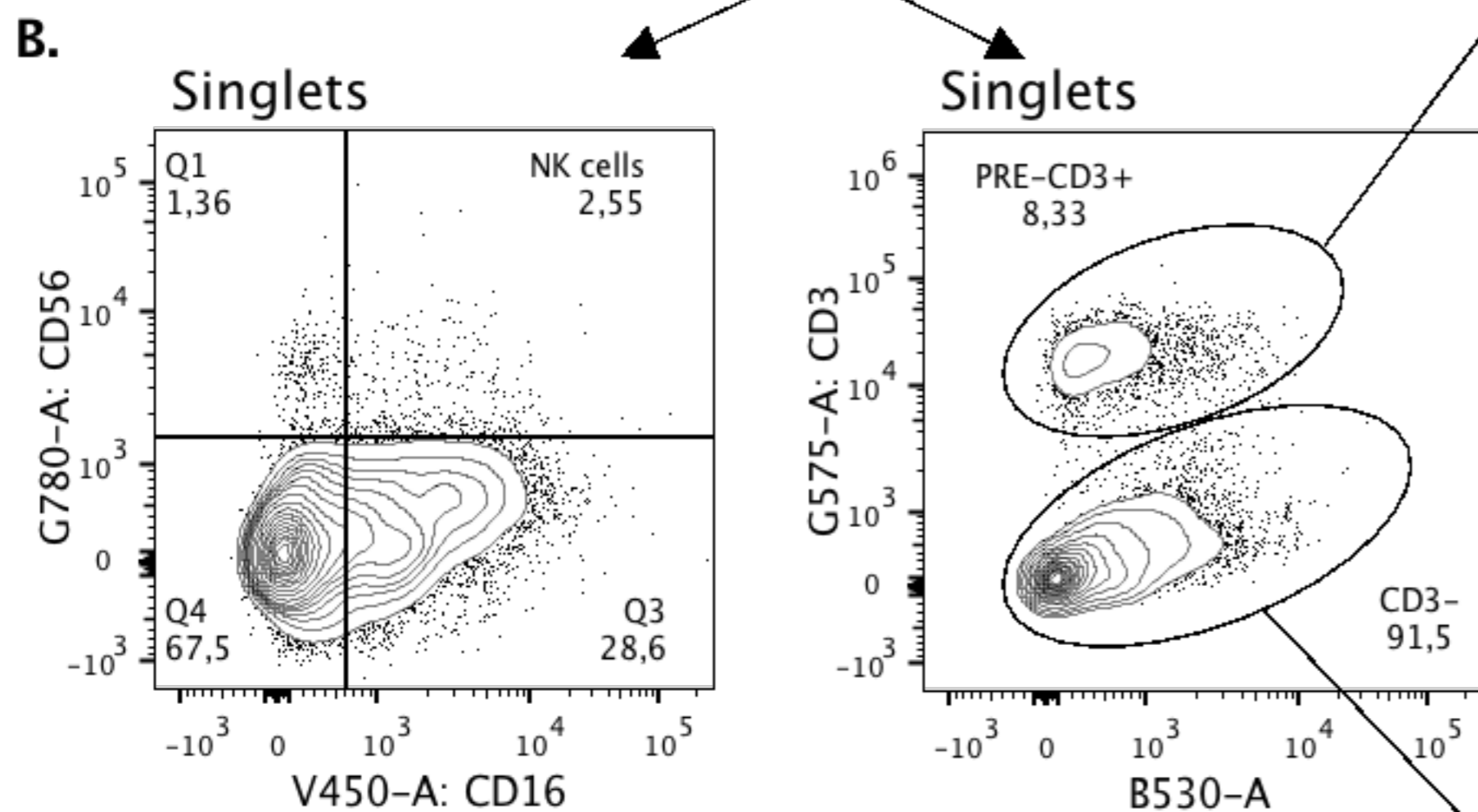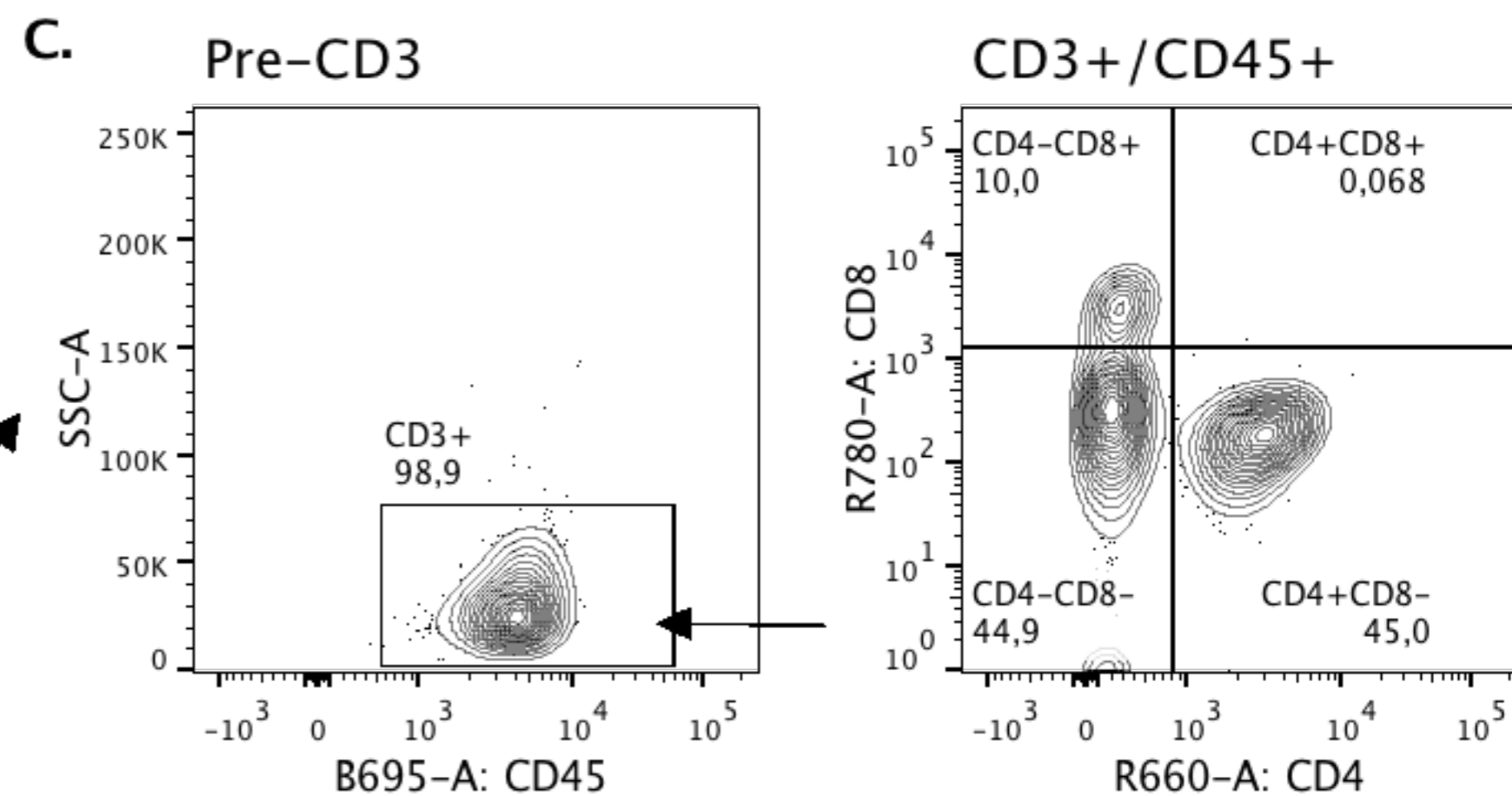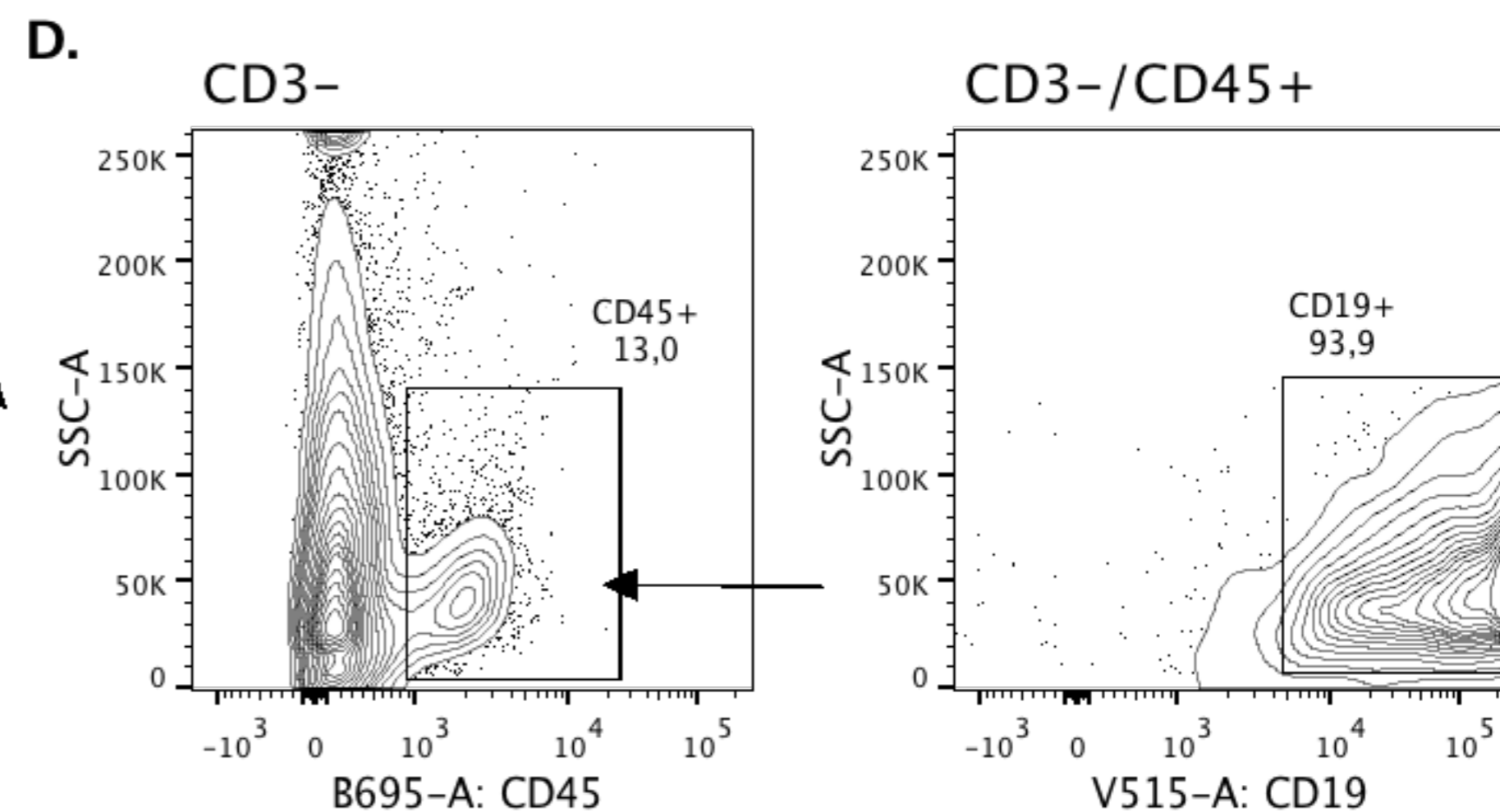

Supplement: Supplementary Figure 1 — Gating strategy to define human splenocyte populations. Splenocytes were labeled with a panel of antibodies in order to discriminate immune relevant splenocyte populations (CD3-PE, CD4-APC, CD8-APC-H7, CD45-PerCP, CD16-V450, CD19-Bv510, CD56-PE-Bio770) and analyzed in a BD LSRFortessa flow cytometer. (A) Singlet cells were gated according to FSC-A/FSC-W. (B) NK cells were determined as CD16+/CD56+ from singlet population. Two other populations were gated according to CD3 staining: PRE-CD3+ and CD3−. (C) PRE-CD3+ cells were confirmed to be lymphocytes by positive CD45 labeling and later classified into the different T cell subsets: CD3+/CD4−/CD8−, CD3+/CD4+/CD8−, CD3+/CD4−/CD8+, or CD3+/CD4+/CD8+. (D) CD3− cells were gated as lymphocytes by CD45+ staining and B cells were stated as CD19+. [file Image1.PDF]
